# Guanylate-binding Protein 1 (Gbp1) Contributes to Cell-autonomous Immunity against *Toxoplasma gondii*

**DOI:** 10.1371/journal.ppat.1003320

**Published:** 2013-04-25

**Authors:** Elizabeth M. Selleck, Sarah J. Fentress, Wandy L. Beatty, Daniel Degrandi, Klaus Pfeffer, Herbert W. Virgin, John D. MacMicking, L. David Sibley

**Affiliations:** 1 Department of Molecular Microbiology, Washington University School of Medicine, St. Louis, Missouri, United States of America; 2 Institute of Medical Microbiology and Hospital Hygiene, Heinrich-Heine-University, Dusseldorf, Germany; 3 Department of Pathology and Immunology, Washington University School of Medicine, St. Louis, Missouri, United States of America; 4 Department of Microbial Pathogenesis, Boyer Centre for Molecular Medicine, Yale University School of Medicine, New Haven, Connecticut, United States of America; University of Medicine and Dentistry of New Jersey, United States of America

## Abstract

IFN-γ activates cells to restrict intracellular pathogens by upregulating cellular effectors including the p65 family of guanylate-binding proteins (GBPs). Here we test the role of Gbp1 in the IFN-γ-dependent control of *T. gondii* in the mouse model. Virulent strains of *T. gondii* avoided recruitment of Gbp1 to the parasitophorous vacuole in a strain-dependent manner that was mediated by the parasite virulence factors ROP18, an active serine/threonine kinase, and the pseudokinase ROP5. Increased recruitment of Gbp1 to Δ*rop18* or Δ*rop5* parasites was associated with clearance in IFN-γ-activated macrophages *in vitro*, a process dependent on the autophagy protein Atg5. The increased susceptibility of Δ*rop18* mutants in IFN-γ-activated macrophages was reverted in Gbp1^−/−^ cells, and decreased virulence of this mutant was compensated in Gbp1^−/−^ mice, which were also more susceptible to challenge with type II strain parasites of intermediate virulence. These findings demonstrate that Gbp1 plays an important role in the IFN-γ-dependent, cell-autonomous control of toxoplasmosis and predict a broader role for this protein in host defense.

## Author Summary

Emerging evidence suggests that the p65 family of guanylate-binding proteins (GBPs), which is upregulated by interferon gamma, play an important role in host defense against intracellular pathogens. We demonstrate that the ability of virulent strains of *Toxoplasma gondii* to avoid recruitment of mouse Gbp1 is mediated by two parasite virulence factors; the serine threonine kinase ROP18 and the pseudokinase ROP5, which controls its activity. GBP proteins required the autophagy protein Atg5 for proper cellular trafficking, recruitment to parasite-containing vacuoles, and pathogen control, strengthening the link between innate immunity and autophagy. The attenuation of mutants lacking ROP18, which show increased susceptibility to clearance by macrophages and decreased virulence in mice, was reverted by deletion of Gbp1, indicating this host factor is needed for resistance to *T. gondii*. Collectively, these findings demonstrate a key molecular interaction between host defenses mediated by GBPs and parasite virulence factors that thwart innate immunity. As GBPs are phylogenetically conserved among vertebrates, including humans, they likely play a broader role in host resistance.

## Introduction

*Toxoplasma gondii* is an apicomplexan protozoan parasite with a broad host range that is capable of causing significant disease in humans and animals [1]. Many wild or domestic animals serve as intermediate hosts, becoming infected either by ingestion of oocysts shed by cats [1], or by carnivorous/omnivorous feeding that facilitates transmission [2]. Human toxoplasmosis is therefore zoonotic, with infection caused by ingestion of tissue cysts in undercooked meat or oocysts that may contaminate food or water [3], [4]. Given the central role of the mouse in the completion of the life cycle of *T. gondii*, understanding the mechanisms of immune control in the mouse are relevant to human infection and may also identify pathways important in human resistance.

Within North America and Europe, strains of *T. gondii* are largely comprised of one of three highly clonal genotypes, referred to as type I, II, and III [5]. These genotypes have highly different phenotypes in the laboratory mice, with type I strains being acutely virulent, type II strains having intermediate virulence, while type III strains are essentially avirulent [5]. Previous genetic crosses have revealed that these differences are due to a small number of polymorphic serine/threonine kinases that are secreted from the rhoptries (ROPs) into the host cell [6]. Among these ROP18 was identified based on the large genetic contribution it makes to differences in acute virulence between highly virulent type I, intermediate virulence type II, and avirulent type III strains [7], [8]. A second locus that contributes more substantially to acute virulence differences between these strains types encodes a polymorphic family of pseudokinases called ROP5 [9], [10]. Collectively, these two loci account for the major strain differences in virulence in the murine model, although other loci have also been implicated in pathogenesis [6].

Resistance to infection with *T. gondii* is largely mediated by IL-12 [11] driving expression of IFN-γ, which activates both toxoplasmastatic and toxoplasmacidal mechanisms [12], in both hematopoietic and non-hematopoietic cells [13]. The primary mechanism of cell-autonomous killing in the mouse is due to IFN-γ induced expression of immunity-related GTPases (IRGs) [14], which are essential for control of infection in macrophages *in vitro* [15], and during *in vivo* infection with type II strains of *T. gondii* [16], [17]. IRG-mediated clearance involves the cooperative recruitment and loading of the GTP-bound IRGs onto the parasitophorous vacuolar membrane (PVM) surrounding the parasite, with subsequent vesiculation and rupture of the vacuole, and destruction of the parasite [18], [19], [20]. A subset of IRG proteins, known as IRGM proteins modulate the activation state of the effector IRG proteins [21]: absence of these IRGM proteins causes spontaneous activation of IRG effectors that form aggregates, compromising their ability to combat pathogens [14]. Similarly, cells lacking Atg5 also show disruption in the function of IRG proteins, which accumulate as GTP-bound forms in cytoplasmic aggregates, and hence fail to clear susceptible strains of *T. gondii* [22], [23]. The accumulation of IRGs on the PVM and the clearance of parasites in macrophages are blocked by ROP18, which phosphorylates several IRG proteins, thus preventing their association with the PVM [24], [25].

Although IRGs are expanded in rodents, they are absent or numerically reduced in many vertebrates including humans, while all vertebrate groups express members of another interferon-inducible gene family, the p65 guanylate-binding proteins (GBPs) [14], [26]. GBPs are structurally related to the dynamins and another known antiviral protein family, the Mx proteins. GBPs range in size from 65–73-kDa and account for over 20% of the proteins induced after IFN-γ treatment [26], [27]. The human genome encodes seven GBPs, while the mouse contains 13 GBPs including two alternative splice isoforms [28], [29]. It has been reported that type I *T. gondii* parasites do not accumulate Gbp1 or Gbp2 on their PVM, while a large percent of both type II and type III parasites show accumulation of these proteins [30], [31], although the molecular basis for this is unknown. Recent work has shown that a deletion of a cluster of GBPs on chromosome 3, including Gbp1, 2, 3, 5, 7 and the splice variant Gbp2ps, increases susceptibility to type II parasites both *in vivo* and *in vitro* [32]. Loss of Gbp1 or Gbp5 reduced the ability of mice to resist *Listeria* and *Mycobacteria* infection [28], [33], and loss of Gbp2 leads to susceptibility to *T. gondii* [34]; however, the role of other individual GBPs in the control of *T. gondii* has not been explored *in vivo*.

Here we explored the role of Gbp1 in cell-autonomous resistance to *T. gondii* and probed the interaction between known parasite virulence factors, ROP5 and ROP18, and the GBP pathway. We also investigate the role for the autophagy protein Atg5 in the homeostasis and function of GBPs and their interdependence on the IRG system in controlling resistance to infection with *T. gondii*.

## Results

### Gbp1 is recruited to vacuoles containing *T. gondii* in ROP5-ROP18 dependent manner

**Figure 1:**
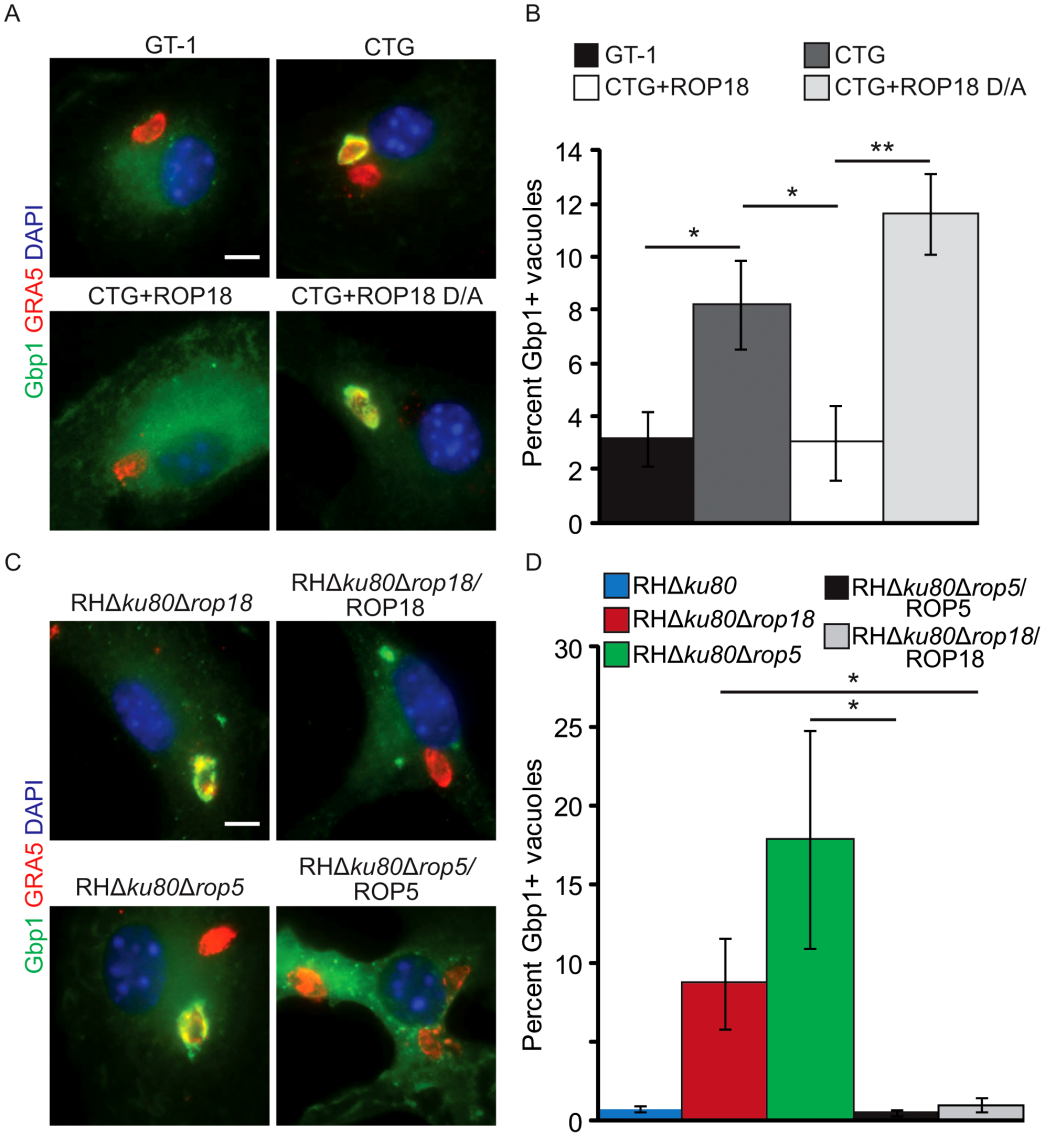
Gbp1 is recruited to vacuoles containing *T. gondii* in a ROP5 and ROP18-dependent manner. (A) Immunofluorescence localization of Gbp1 on the PVM in IFN-γ-activated BMM (10 U/ml IFN-γ, 0.1 ng/ml LPS). Images shown are 2 hr post infection with GT-1 (type I), CTG (type III), CTG+ROP18 (type III expressing ROP18) and CTG+ROP18 D/A (type III expressing kinase-dead ROP18) parasites. Gbp1 was localized with rabbit polyclonal sera followed by goat anti-rabbit IgG conjugated to Alexa Fluor 488. The vacuole marker GRA5 was detected with mAb Tg 17–113 followed by goat anti-rabbit IgG conjugated Alexa Fluor 594. Scale bar = 5 µm. (B) Quantification of Gbp1 localization to the PVM surrounding the indicated parasite strains in IFN-γ-activated BMMs examined at 2 hr post infection. (C) Immunofluorescence localization of Gbp1 on the PVM in IFN-γ-activated BMM (100 U/ml IFN-γ, 0.1 ng/ml LPS) examined at 2 hr post infection. Comparison of type I parasites lacking ROP18 (RHΔ*ku80*Δ*rop18*) or ROP5 (RHΔ*ku80*Δ*rop5*) *vs.* the respective complemented strains (RHΔ*ku80*Δ*rop18/*ROP18) and (RHΔ*ku80*Δ*rop5/*ROP5). Gbp1 was localized with rabbit polyclonal sera followed by goat anti-rabbit IgG conjugated to Alexa Fluor 488. The vacuole marker GRA5 was detected with a mAb Tg 17–113 followed by goat anti-rabbit IgG conjugated Alexa Fluor 594. (D) Quantification of Gbp1 localization to the PVM in IFN-γ-activated BMMs macrophages examined at 2 hr post infection. Strains indicated in (A) compared to the type I parent (RHΔ*ku80*). Mean ± S.D.P., n = 3 experiments. (*P<0.05, Student's *t* test).

To determine whether the recruitment of GBPs to the PVM surrounding intracellular parasite is blocked in a ROP18-dependent manner similar to IRGs, we localized Gbp1 in IFN-γ-activated bone marrow derived macrophages (BMM). The influence of ROP18 on GBP recruitment was examined using a previously described transgenic parasites that express ROP18_I_ in the type III background [8]. Type I parasites (i.e. GT-1 strain) largely prevented recruitment of Gbp1 (Fig. 1A,B). Similarly, type III parasites expressing a kinase-active version of ROP18 (i.e. CTG+ROP18) avoided accumulation of Gbp1 over the first two hr post infection (Fig. 1A,B). In contrast, a significantly higher percent of type III vacuoles (i.e. CTG strain) were positively stained for Gbp1 on the PVM (Fig. 1A,B). The kinase activity of ROP18 was required to prevent Gbp1 recruitment, as expression of a kinase dead ROP18 (i.e. CTG+ROP18 D/A) did not prevent recruitment to the PVM (Fig. 1A,B).

In addition to the active kinase ROP18, it has recently been shown that the pseudokinase ROP5 is important for acute virulence of *T. gondii* [10], [35]. Therefore, we examined the role of these two rhoptry proteins in the prevention of Gbp1 recruitment to the PVM using a loss-of-function approach. Recruitment of Gbp1 was monitored in IFN-γ-activated BMM infected with type I parasites lacking ROP18 (RHΔ*ku80*Δ*rop18*) or ROP5 (RHΔ*ku80*Δ*rop5*), *vs.* the respective complemented strains (RHΔ*ku80*Δ*rop18*/ROP18 and RHΔ*ku80*Δ*rop5/*ROP5) and the wild type strain (RHΔ*ku80*) (Fig. 1C,D). Wild type or complemented parasites expressing ROP18 and ROP5 essentially prevented Gbp1 accumulation on the PVM, while Δ*rop18* parasites showed significantly higher Gbp1 accumulation. Interestingly, Δ*rop5* parasites showed the highest level of Gbp1 recruitment (Fig. 1C,D). Collectively these data indicate that known *T. gondii* virulence factors ROP18 and ROP5 are necessary to prevent Gbp1 accumulation on the PVM surrounding parasites in IFN-γ-activated macrophages.

### Gbp1 recruitment to the parasite containing vacuole is associated with vesiculation and rupture

**Figure 2:**
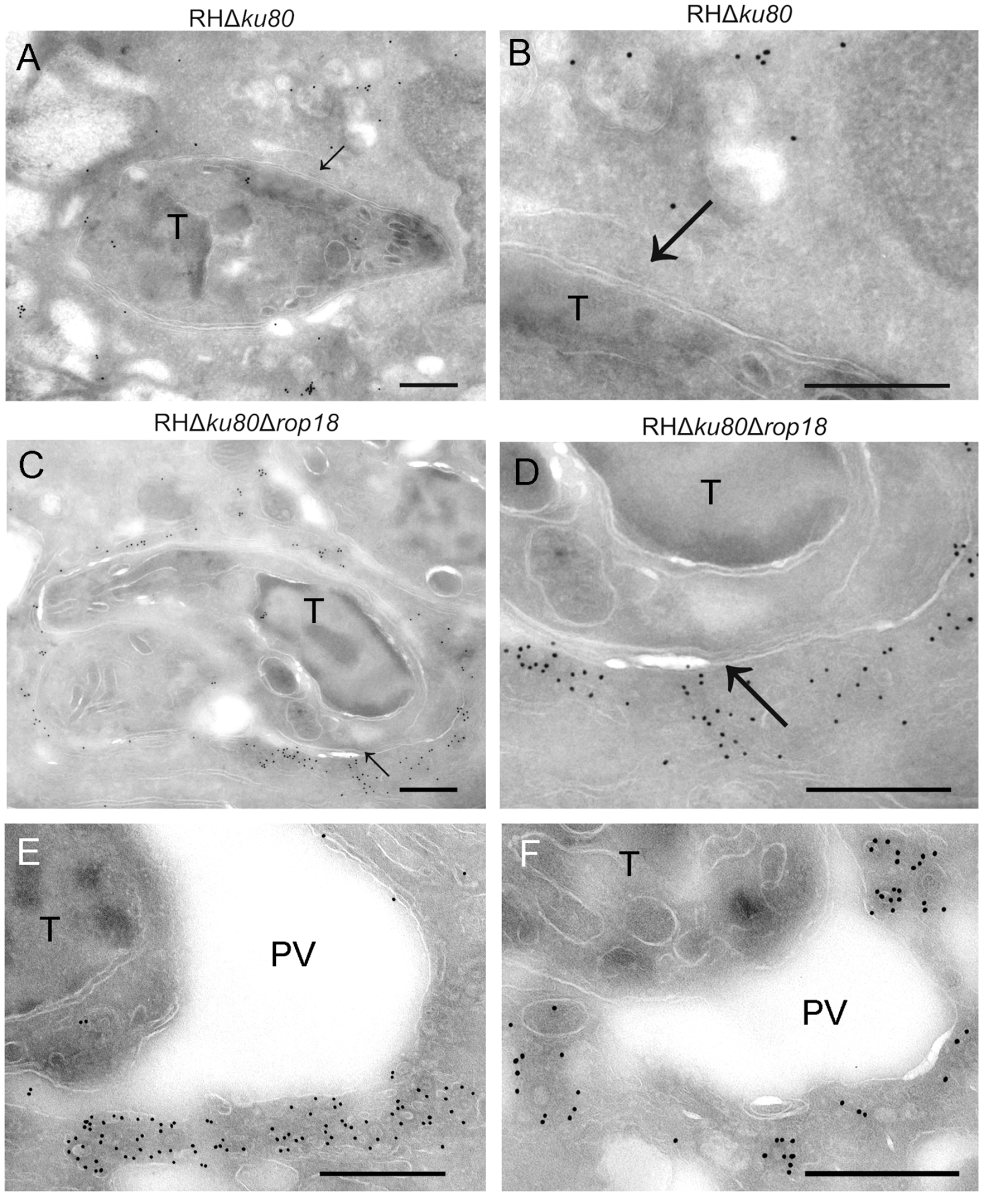
Recruitment of Gbp1 to the parasitophorous vacuole membrane is associated with vesiculation. Cryo-immuno-EM localization of Gbp1 in IFN-γ-activated RAW 264.7 macrophages (10 U/ml IFN-γ, 0.1 ng/ml LPS). (A) Distribution of Gbp1 in activated macrophages infected with wild type *T. gondii* (T) parasites (RH*Δku80*). Arrow indicates PVM. (B) Enlargement of A showing PVM (arrow). (C–F) Distribution of Gbp1 in IFN-γ-activated macrophages infected with ROP18-deficient parasites (RH*Δku80Δrop18*). Arrow indicates PVM. (D) Enlargement of C showing PVM (arrow). (E,F) Distribution of Gbp1 in membrane vesicles in the vicinity of the PVM. Cells were fixed at 2 hr post-infection and immunostained using rabbit anti-Gbp1 (Rab α Gbp1) polyclonal sera followed by goat anti-rabbit IgG conjugated to 18 nm gold. Scale bars = 500 nm.

IRG proteins have previously been shown to localize to the PVM surrounding susceptible *T. gondii* parasites [18], [19], [24], although similar findings have not been reported for GBPs. Cryo-immuno electron microscopy (EM) of IFN-γ-activated RAW 264.7 macrophages revealed that Gbp1 was localized diffusely in the cytosol of cells infected with wild type parasites, and not on the PVM (Fig. 2A,B). In contrast, Gbp1 strongly localized in the vicinity of the PV in cells infected with Δ*rop18* parasites (Fig. 2C,D). Although limited staining was observed on the PVM, Gbp1 was associated with nearby membrane vesicles that clustered around the vacuole (Fig. 2E,F). The prominent localization of Gbp1 to vesicles surrounding the PVM suggests that it may be involved in delivery of other components to the compartment, or disposal of membrane following vesiculation and vacuole membrane rupture. Gbp1 positive vesicles also collect around phagosomes containing mycobacteria in IFN-γ-activated macrophages [28], suggesting that Gbp1 recruitment could serve as a common protective mechanism against different pathogen classes.

**Figure 3:**
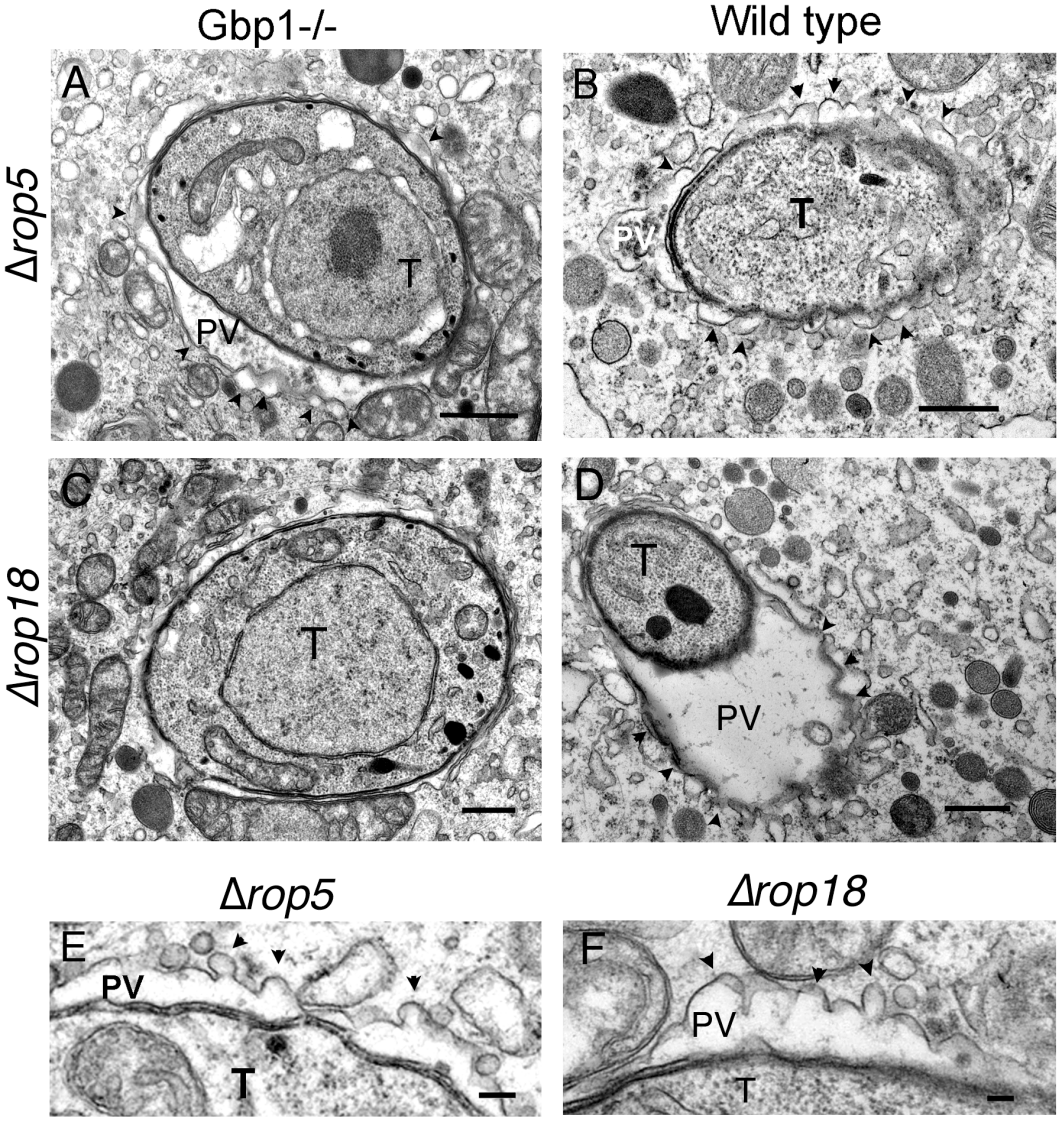
Ultrastructural features of the parasitophorous vacuole membrane in IFN-γ-activated cells. (A–D) Ultrastructural features of the parasitophorous vacuole (PV) *T. gondii* (T) in IFN-γ-activated (100 U/ml IFN-γ, 0.1 ng/ml LPS) BMM from Gbp1^−/−^ or wild type mice infected with ROP5-deficient (*Δrop5*) or ROP18-deficient (*Δrop18*) parasites. (E–F) Enlargement of the PV surrounding parasites in IFN-γ-activated BMM from wild type mice shows the highly scalloped appearance of the membrane (marked by black arrowheads). Scale bars = 500 nm in A–D, 100 nm in E,F.

We also examined the morphological features of the PVM surrounding susceptible parasites by conventional EM (Fig. 3 A–F). Vacuoles containing ROP5-deficient (Fig. 3B) or ROP18-deficient (Fig. 3D) parasites in IFN-γ-activated BMM from wild type mice were slightly distended with an enlarged lumen and showed marked vesiculation, membrane blebbing, and accumulation of small vesicles around the PV. In addition, both ROP5 and ROP18 deficient parasites showed evidence of vacuole rupture, leaving the parasite free in the cytosol, where it often underwent degradation (Fig. S2). In Gbp1^−/−^ BMM cells activated with IFN-γ, vacuoles containing ROP5-deficient parasites also showed frequent vesiculation accompanied by an enlarged lumen (Fig. 3A), while ROP18-deficient (Fig. 3C) parasites were found in more a closely-fitting vacuole surrounded by host cell mitochondria and which had a smooth circumference, characteristic of an intact PV. Enlargement of the PVM revealed that membrane blebbing around both ROP5 or ROP18-deficient parasites in wild type cells occurred with a marked curvature and regular scalloped pattern (Fig. 3 E,F). These features are highly reminiscent of the previously described vesiculation of the PV membrane that accompanies IRG-recruitment to the PVM and vacuole destruction [18], [23], [24]. Notably, this process is interrupted the Gbp1^−/−^ cells, at least in the terms of the fate of ROP18-deficient parasites, a result consistent with the survival *vs.* clearance of ROP5 or ROP18-deficient parasites in IFN-γ-activated BMM from wild type *vs.* Gbp1^−/−^ mice, as described below.

### Gbp1 localization is altered in Atg5-deficient cells

**Figure 4:**
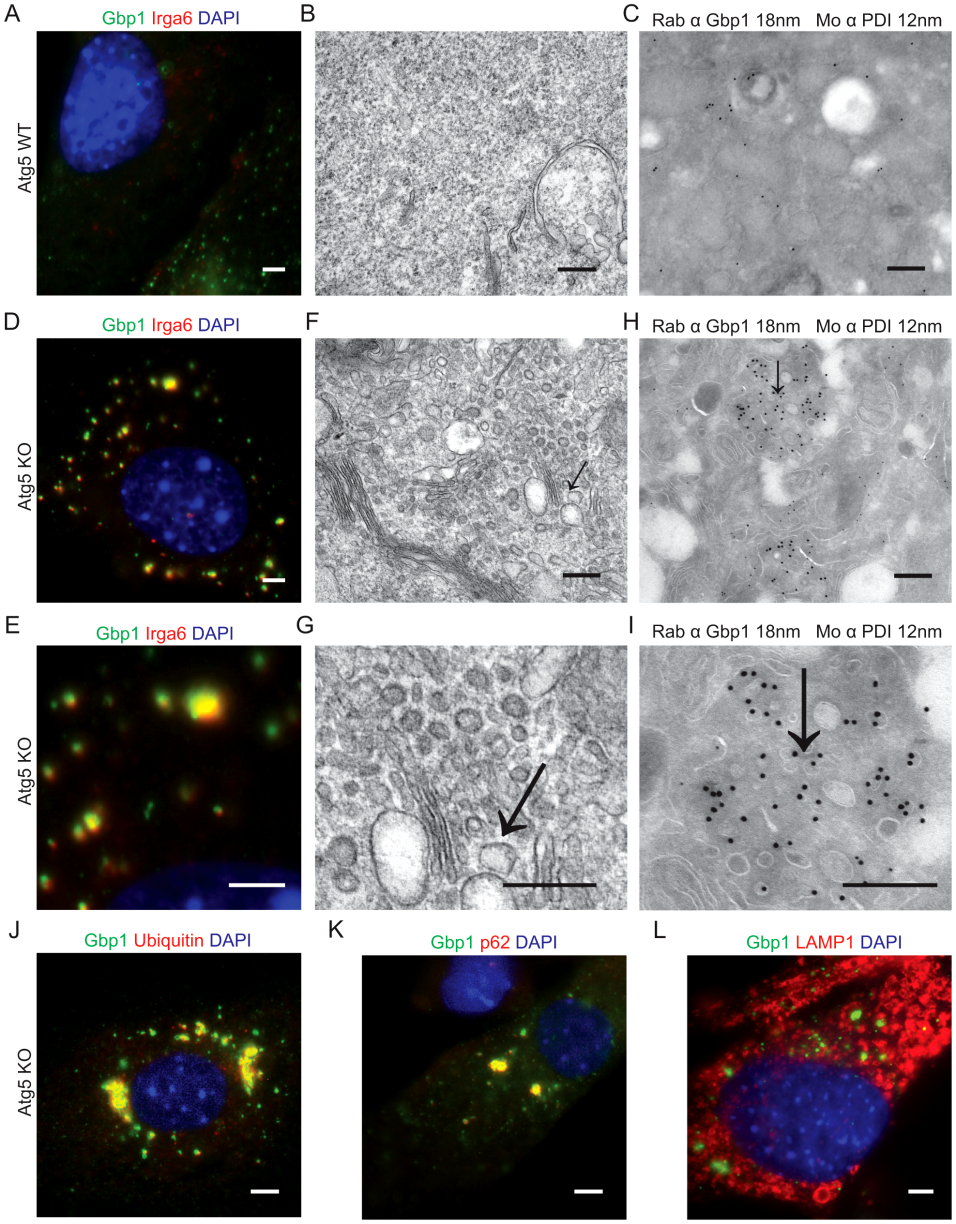
Gbp1 localization is altered in Atg5-deficient cells. Immunofluorescence localization of Gbp1 and Irga6 in wild type (WT) (A) or Atg5-deficient (Atg5 KO) (D,E) MEFs activated with IFN-γ (10 U/ml IFN-γ, 0.1 ng/ml LPS). Gbp1 was localized with rabbit polyclonal sera followed by goat anti-rabbit IgG conjugated to Alexa fluor 488. Irga6 was localized with mAb 10D7 followed by goat anti-mouse IgG conjugated to Alexa fluor 594. Transmission electron microscopy of wild type (WT) (B) or Atg5-deficient (Atg5 KO) (F,G) IFN-γ-activated MEFs. Scale bar = 200 nm. Cryo-immuno-EM of IFN-γ-activated wild type (WT) (C), or Atg5-deficient (Atg5 KO) (H,I), MEFs. Gbp1 was detected with rabbit polyclonal sera (Rab α Gbp1) followed by goat-anti-rabbit IgG conjugated to 18 nm gold beads and protein disulfide isomerase using mAb antibody 1D3 (Mo α PDI) followed by goat anti-mouse IgG conjugated to 12 nm gold beads. Scale bar = 200 nm. Immunofluorescence of Gbp1 co-localized with p62 (J), ubiquitin (K), or LAMP1 (L) in IFN-γ-activated MEFs from Atg5-deficient mice (Atg5KO). Gbp1 was localized with rabbit polyclonal sera followed by goat anti-rabbit IgG conjugated to Alexa fluor 488. p62 was localized with guinea pig polyclonal sera followed by goat anti-guinea pig IgG conjugated to Alexa Fluor 594. Ubiquitin was localized with mAbFK2 followed by goat anti-mouse IgG conjugated to Alexa fluor 594. LAMP1 was localized with rat mAB 1D4B followed by goat anti-rat 1gG conjugated to Alexa fluor 594. Image in L is a deconvolved Z slice, all other fluorescence images are wide field. Scale bars = 5 µm.

The autophagy protein Atg5 is required for resistance to type II parasite infection in mice [23], in part due to the fact that in its absence, members of the IRG family are mislocalized into large aggregates in the host cell and hence are not recruited to parasite-containing vacuoles [22], [23]. To examine the role for Atg5 in the localization of Gbp1, we stained IFN-γ-activated Atg5-deficient (Atg5 KO) and wild type (Atg5 WT) mouse embryonic fibroblasts (MEFs) for Gbp1 and Irga6. In wild type cells, Irga6 showed a diffuse cytoplasmic distribution (Fig. 4A) and these cells exhibited a normal homogenous cytosol where Gbp1 was distributed in small clusters, as shown by cryo-immuno EM (Fig. 4B,C). In contrast, in the absence of Atg5, Gbp1 was localized to large aggregates within the cell, and these structures partially co-localized with aggregates of Irga6 that was in the GTP-bound state (as detected with a conformation specific antibody) (Fig. 4D,E). Transmission electron microscopy showed large accumulations of vesicles in the cytoplasm of Atg5-deficient cells (Fig. 4F,G), which were absent in the wild type cells (Fig. 3B). Cryo-immuno EM revealed an accumulation of Gbp1 in clusters of vesicles that accumulated in the Atg5-deficient cells (Fig. 4H). Gbp1 positive vesicles were distinct from the endoplasmic reticulum (ER), although they were interspersed with ER membranes, as indicated by staining for protein disulfide isomerase (PDI) (Fig. 4H,I).

To further assess the composition of the Gbp1 aggregates in the Atg5-deficient (Atg5 KO) MEFs, we examined their colocalization with ubiquitin and p62, which were first reported to localize with Gbp1 in uninfected and mycobacterium-infected macrophages [28], and also implicated in aggregates of Gbp2 that form in the absence of IRGM proteins [36]. Immunofluorescence labeling demonstrated that Gbp1 aggregates largely co-localize with p62 and ubiquitin (Fig. 4J,K); however, the aggregates were LAMP1 negative (Fig. 4L). In comparison, the rare aggregates that also normally form in wild type cells were typically surrounded by LAMP1 positive vesicles, suggesting they fuse with lysosomes and are degraded (Fig. S1). Consistent with this model, the frequency of IRG-GBP aggregates was substantially increased by treatment with bafilomycin, which blocks lysosome fusion (Fig. S1). In bafilomycin treated cells ∼20% of cells showed aggregates of Gbp1 and Irga6 compared to ∼1% of DMSO treated cells.

### Atg5 is required for the recruitment of Irgb6 and Gbp1 to parasitophorous vacuoles and clearance *in vitro*

**Figure 5:**
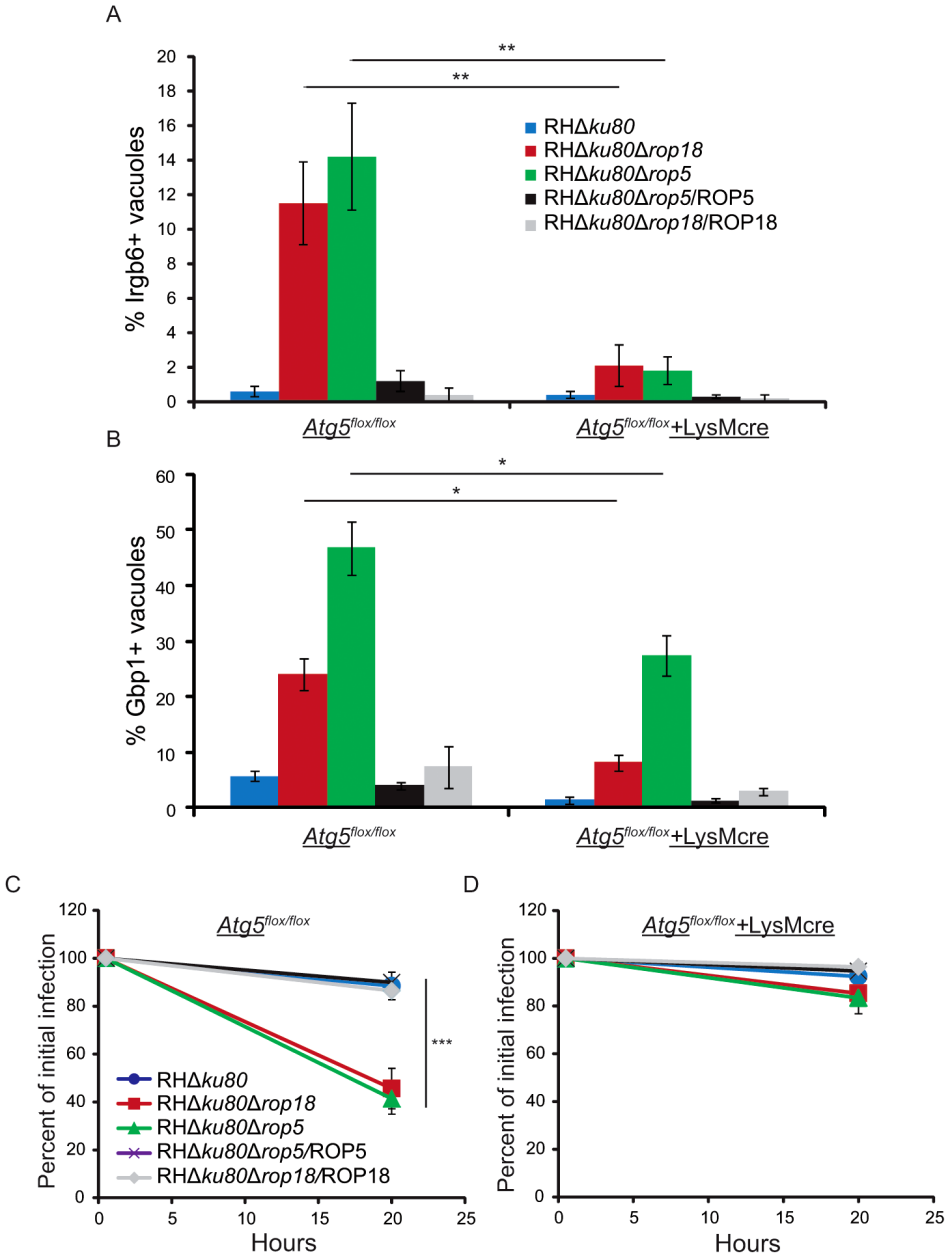
Cells lacking Atg5 are unable to recruit Irgb6 or Gbp1 to the parasitophorous vacuole or clear susceptible parasites. (A) Quantification of Irgb6 localization to the parasitophorous vacuoles surrounding intracellular parasites in IFN-γ-activated BMM (50 U/ml IFN-γ, 0.1 ng/ml LPS) from *Atg5^flox/flox^* or *Atg5^flox/flox^+*LysMCre mice. Means ± S.D., n = 6 samples from 2 combined experiments. (**P<0.005, Student's *t* test). (B) Quantification of Gbp1 recruitment to the parasitophorous vacuoles surrounding intracellular parasites IFN-γ-activated BMM (100 U/ml IFN-γ, 0.1 ng/ml LPS) from *Atg5^flox/flox^* or *Atg5^flox/flox^*+LysMcre mice. Representative experiment from three independent experiments with similar outcomes. Mean ± S.D., n = 3 replicates per group. (*P<0.05, Student's *t* test). *In vitro* clearance of parasites in IFN-γ-activated BMM (50 U/ml IFN-γ, 0.1 ng/ml LPS) from *Atg5^flox/flox^* (C) or *Atg5^flox/flox^*+LysMcre (D) mice. Means ± S.D., n = 6 samples from 2 combined experiments. (*** *P*<0.001, Student's *t* test).

The altered distribution of Gbp1 and Irga6 in Atg5-deficient MEFs suggested that recruitment of other IRG members, such as Irgb6, to the PVM of susceptible parasites might be impaired. To examine the role of Atg5 in recruitment of Irgb6 and Gbp1 in BMM, we took advantage of the previously described conditional deletion strain *Atg5^flox/flox^*+LysMcre, in which Atg5 is specifically ablated in myeloid cells [23]. In IFN-γ-activated BMM with functional Atg5 (*Atg5^flox/flox^*), Δ*rop18* or Δ*rop5* parasites showed increased recruitment of Irgb6 (Fig. 5A) and Gbp1 (Fig. 5B) compared to the type I wild type or the ROP18 and ROP5 complemented strains. In contrast, BMM lacking functional Atg5 (*Atg5^flox/flox^*+LysMcre) showed significantly reduced Irgb6 (Fig. 4A) and Gbp1 (Fig. 5B) recruitment to all strains. This reversal in accumulation was particularly evident for Δ*rop18* and Δ*rop5* parasites, which normally show elevated accumulation of Irgb6 [24] and Gbp1 (Fig. 1B). Infection of IFN-γ-activated macrophages revealed that both Δ*rop18* and Δr*op5* parasites underwent enhanced clearance in wild type cells (*Atg5^flox/flox^*) (Fig. 5C). This decrease in survival was reverted to normal in Atg5-deficient macrophages (*Atg5^flox/flox^*+LysMcre) (Fig. 5D). Together, these data indicate that Atg5 plays an important role in homeostasis of Irgb6 and Gbp1 and in its absence, recruitment of these effectors to the PVM surrounding susceptible parasites is compromised, preventing parasite clearance in IFN-γ-activated macrophages.

### Gbp1^−/−^ cells are defective in parasite clearance and Irgb6 localization to the PV

**Figure 6:**
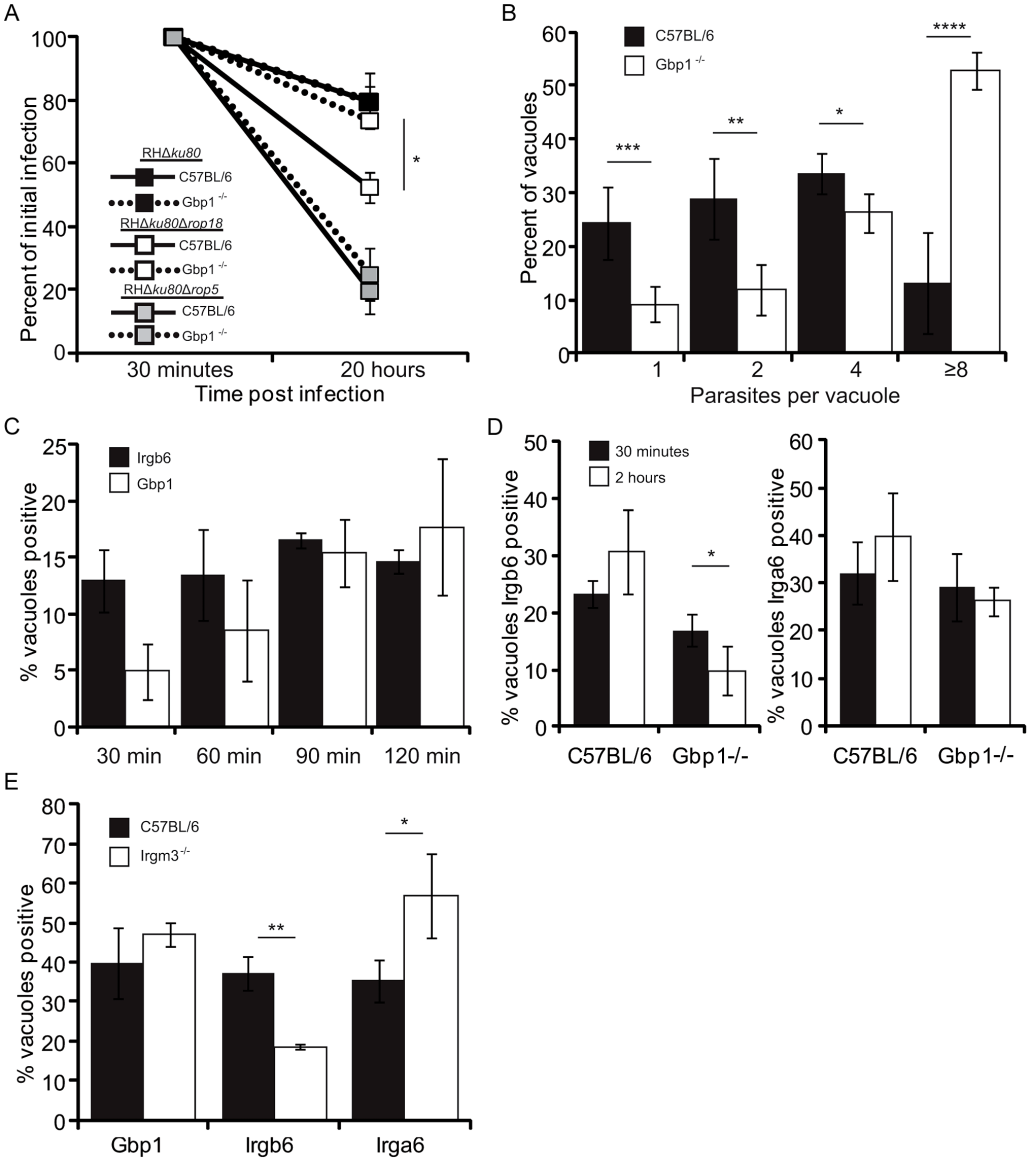
Gbp1 is necessary for control of *T. gondii*. (A) *In vitro* clearance of ROP18-deficient (RHΔ*ku80*Δ*rop18*), ROP5-deficient (RHΔ*ku80*Δ*rop5*) and wild type (RHΔ*ku80*) *T. gondii* in IFN-γ-activated BMM (50 U/ml IFN-γ, 10 ng/ml LPS) from Gbp1^−/−^ or C57BL/6 mice. Parasites were visualized using mAb to SAG1 (DG52) directly conjugated to Alexa Fluor 594. Percent of cells infected at 20 hr compared to 30 min time point. Mean ± S.D.P., n = 3–4 experiments (*P<0.005, Student's *t* test). (B) Analysis of replication in IFN-γ-activated BMM (as in A) infected with wild type (RHΔku80) parasites and analyzed at 20 hr post-infection. Mean number of parasites per vacuole ± S.D. n = 2 (*P<0.05, **P<0.005, ***P<0.0005, **** *P*<0.000005, Student's *t* test). (C) Quantification of Irgb6 and Gbp1 localization to parasitophorous vacuoles of intracellular parasites in IFN-γ-activated BMM (100 U/ml IFN-γ, 0.1 ng/ml LPS) infected with ROP5-deficient (RHΔ*ku80*Δ*rop5*) parasites. Mean ± S.D. n = 6 samples from 2 combined experiments. (D) Quantification of Irgb6 and Irga6 localization to parasitophorous vacuoles surrounding ROP18-deficient (RHΔ*ku80*Δ*rop18*) parasites in C57BL/6 vs. Gbp1^−/−^ BMM activated with 100 U/ml IFN-γ and 0.1 ng/ml LPS. Cells were infected for 30 min *vs.* 2 hr, fixed, permeabilized and stained using rabbit anti-Irgb6 and mAb against GRA5, or mAb 10D7 against Irga6 and rabbit anti-GRA7, and detected using corresponding Alexa Flour secondary antibodies. Representative of two experiments with similar outcomes. Mean ± S.D.P, n = 4. (*P<0.05, Student's *t* test). (E) Quantification of Gbp1, Irgb6 and Irga6 localization to parasitophorous vacuoles of ROP5-deficient parasites (RHΔ*ku80*Δ*rop5*) in C57BL/6 *vs.* Irgm3^−/−^ BMM activated with 100 U/ml IFN-γ and 0.1 ng/ml LPS. Representative experiment from 2–3 independent experiments with similar outcomes. Mean ± S.D., n = 3 replicates per group. (*P<0.05, **P<0.005, Student's *t* test).

To examine the role of Gbp1 in resistance to *T. gondii*, we first tested the ability of IFN-γ-activated BMM from Gbp1-deficient mice (Gbp1^−/−^) to clear parasites following overnight infection *in vitro*. We infected BMM from C57BL/6 and Gbp1^−/−^ mice with wild type parasites, which largely resist clearance in activated macrophages, and Δ*rop18* parasites, which are cleared by ∼50% in activated macrophages [24]. As expected, C57BL/6 BMM cleared Δ*rop18* parasites to about 50% of the initial infection (Fig. 6A). Strikingly, Gbp1^−/−^ BMM showed a reduced ability to clear susceptible Δ*rop18* parasites following overnight incubation, restoring survival to ∼80%, a level that was similar to wild type parasites in either strain of mice (Fig. 6A). In contrast, the increased clearance of Δr*op5* parasites seen in C57BL/6 BMM was not reversed in Gbp1^−/−^ deficient BMM (Fig. 6A).

Recent studies on the deletion of the Gbp^chr3^ locus in the mouse indicated that the ability of IFN-γ-activated macrophages to prevent replication of intracellular parasites, referred to as stasis, is also compromised in the absence of this locus [32]. Therefore we examined the ability of activated wild type (C57BL/6) or Gbp1^−/−^ BMM to restrict the intracellular replication of *T. gondii* at 20 hr post infection. Under the activation conditions used here, the majority of intracellular *T. gondii* in wild type cells were found in vacuoles containing 1–2 parasites, with a few having replicated to clusters of 4 (Fig. 6B). In contrast, the majority of intracellular *T. gondii* were found in rosettes of 8, with the remainder largely being found in clusters of 4. Although the level of stasis achieved in wild type cells was less than that reported previously [32], the ability of Gbp1^−/−^ BMM to control replication was significantly impaired (Fig. 6B).

To examine the kinetics of IRG *vs.* GBP recruitment, we examined the percentage of PVs containing ROP18 deficient parasites that became visibly positive over the first 2 hr post-infection in BMM. Irgb6 positive vacuoles were elevated at 30 min post-infection and they remained at similar levels during the first 120 min (Fig. 6C). In contrast, accumulation of Gbp1 was delayed: the percentage of positive vacuoles was initially lower at 30 min and only plateaued at 90–120 min (Fig. 6C). Over this time course, the majority of vacuoles became positive for both Irgb6 and Gbp1 (64.7±4.2%), while most of the remaining vacuoles stained only with Gbp1 (33.8±5.8%), and only a minority being Irgb6 positive only (2±2%). Combined with the differences in kinetics, these findings suggest that Gbp1 is recruited after Irgb6, and that it remains on the vacuole after Irgb6 is recycled. Alternatively, Gbp1 may have additional IRG-independent mechanisms to target the PV, as discussed below. Although the percentage of Irgb6 positive vacuoles remained the same in Gbp1^−/−^ cells at 30 min, it was significantly reduced at 2 hr when compared to wild type cells (C57BL/6) (Fig. 6D). In contrast, Irga6 positive PV increased slightly between 30 min and 2 hr in wild type and did not change in Gbp1^−/−^ BMM (Fig. 6D). These findings indicate that Gbp1 influences the recruitment and/or retention of some IRG proteins onto the PVM surrounding susceptible parasites.

To assess the requirement of IRG proteins in the recruitment of Gbp1 to susceptible parasites, we examined accumulation of Gbp1, Irgb6, and Irga6 to Δ*rop5* parasites in Irgm3^−/−^ *vs.* wild type (C57BL/6) BMM at 2 hr post infection. Similar to previous reports showing that Irga6 and Gbp2 partially form aggregates in the cytosol of Irgm3^−/−^ cells [36], we observed that Irga6, Irgb6 and Gbp1 showed focal clusters of staining in Irgm3^−/−^ cells, although a majority of the proteins were still homogenously dispersed when examined by immunofluorescence microscopy (data not shown). Despite the formation of some aggregates, Gbp1 accumulation on the PV was not significantly different in Irgm3^−/−^ *vs.* wild type BMM, while Irga6 positive PV increased in Gbp1^−/−^ knockout cells (Fig. 6E). In contrast, the percent of Irgb6 positive PV was significantly lower in the Irgm3^−/−^ BMM. These data support previous findings that Irgm3 is required for efficient Irgb6 loading onto the PV of susceptible parasites [9], but reveal that is not required for either Irga6 or Gbp1 recruitment. Indirectly this implies that Irgb6 is also not required for Gbp1 recruitment to the PVM. Collectively, these results demonstrate that Gbp1 is critical for the control and clearance of *T. gondii* in IFN-γ-activated BMM *in vitro*, and suggests that it while it works cooperatively with the IRGs, it may not depend on them for recruitment.

### Gbp1-deficient mice are significantly more susceptible to challenge with moderately virulent *T. gondii*

**Figure 7:**
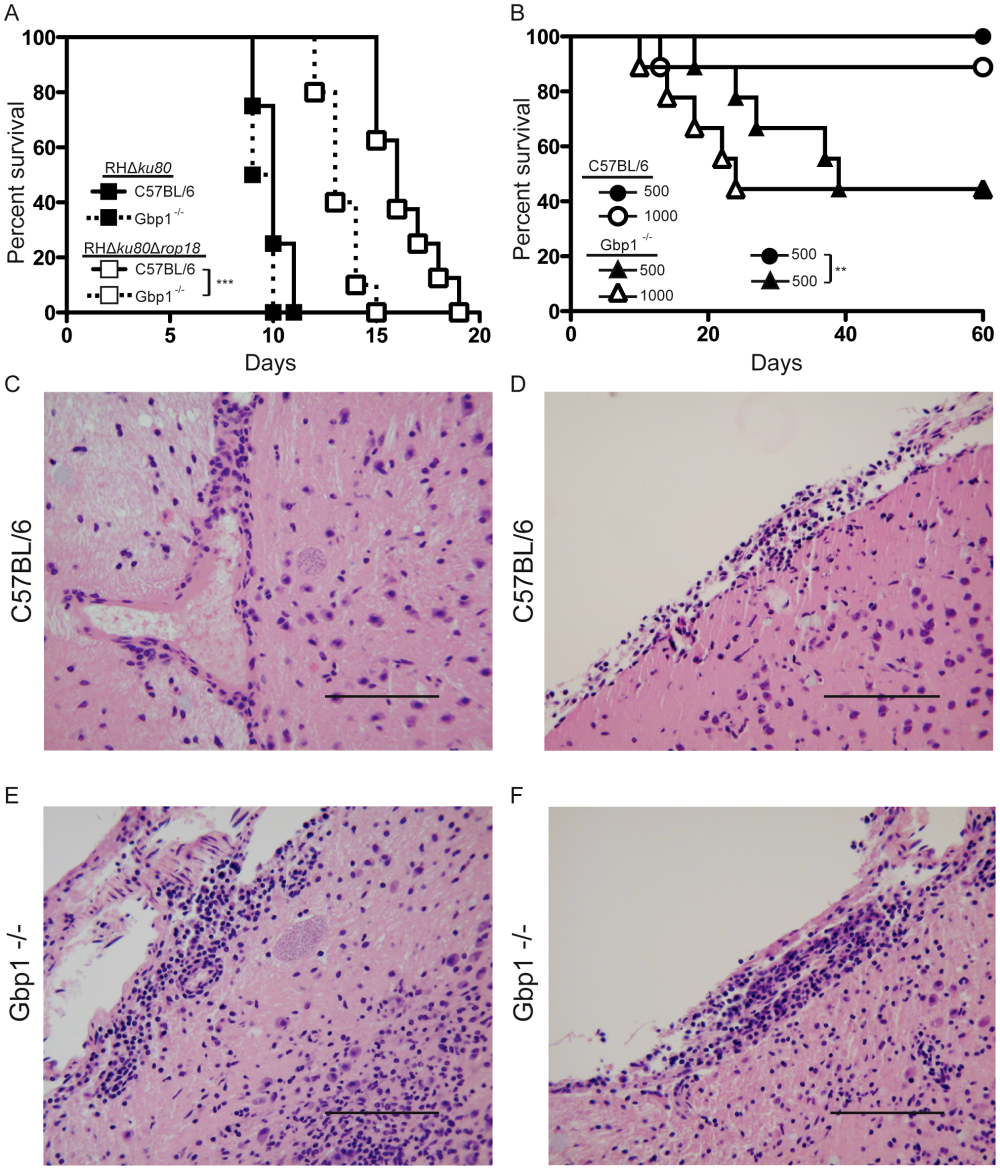
Gbp1-deficient animals are more susceptible to *T. gondii* infection. (A) Survival of Gbp1^−/−^ or C57BL/6 mice s.c. infected with 100 ROP18-deficient (RHΔ*ku80*Δ*rop18*) or wild type (RHΔ*ku80*) parasites. Two independent experiments combined, n = 8 mice per parasite strain for C57BL/6 and n = 10 mice per parasite strain for Gbp1^−/−^. (***P<0.0005, Gehan-Breslow-Wilcoxon Test). (B) Survival of Gbp1^−/−^ or Combination of two experiments where n = 8–9 animals per group. (** *P*<0.005, Gehan-Breslow-Wilcoxon Test). (C–F) H&E paraffin sections from the thalamus region of the brains from representative surviving mice (from B) at 60 days post infection. Scale bars = 100 nm.

Next we wanted to examine how Gbp1 deficiency would affect *in vivo* challenge with parasites. We challenged C57BL/6 and Gbp1^−/−^ mice with highly virulent type I strain RH *vs.* ROP18-deficient parasites and monitored their survival. Δ*rop18* parasites showed a significantly delayed time to death compared with wild type parasites in wild type mice (Fig. 7A). The deficiency of Δ*rop18* parasites was partially reversed in Gbp1^−/−^ mice, which succumbed to challenge 4 days earlier than C57BL/6 mice (Fig. 7A). When challenged with the highly virulent wild type parasites, Gbp1^−/−^ mice showed almost equivalent survival to C57BL/6 mice, although this is not unexpected given the high virulence of type I strains (Fig. 7A). Additionally, the highly attenuated phenotype of the Δ*rop5* parasites in mice was not reversed in Gbp1*^−/−^* mice (data not shown), consistent with the enhanced clearance of this parasite mutant in Gbp1^−/−^ cells *in vitro*, both of which reflect much more severe defect in this mutant, as described previously [9].

To further explore the defect in resistance, we challenged Gbp1^−/−^ and C57BL/6 mice with a moderately virulent type II parasite strain at two different doses and monitored their survival for 60 days. C57BL/6 mice were largely resistant to infection with type II parasites, with a single mouse succumbing to infection at day 13 post infection with 1,000 parasites (Fig. 7B). Gbp1^−/−^ mice were more susceptible to type II parasites with animals starting to succumb to challenge 3 days earlier than C57BL/6 mice. Additionally, significantly fewer Gbp1^−/−^ mice survived challenge with either 500 or 1,000 parasites (Fig. 7B).

The delayed death phenotype of Gbp1^−/−^ mice is reminiscent of type II infection in Nos2^−/−^ mice, which succumb due to encephalitis [37]. Therefore, we examined the brains of surviving C57BL/6 and Gbp1^−/−^ animals at day 60 days post infection for signs of encephalitis by H&E staining. Sections from the brain of an infected C57BL/6 mouse showed mild perivascular cuffing, minimal focal accumulation of lymphocytes, and a single tissue cyst (Fig. 7C, D). Sections from the brain of an infected Gbp1^−/−^ mouse showed more severe pathological changes including moderate focal gliosis, multifocal perivascular cuffing, moderate thickening of the meninges, and multiple tissue cysts (Fig. 7 E,F).

Collectively, these results demonstrate that Gbp1 plays an important role in the control of infection *in vivo* as Gbp1-deficent animals show increased susceptibility both during acute and chronic infection.

## Discussion

IFN-γ plays a crucial role in activating cells to control proliferation and destroy intracellular parasites. Here we demonstrate that Gbp1 plays an important role in this cell-autonomous control *in vitro* and in resistance to *T. gondii* infection *in vivo*. GBPs may work cooperatively with IRGs, which have previously been implicated in resistance to *T. gondii*, and both families of effectors rely on Atg5 for homeostasis. Our work underscores the importance of the GBPs in resistance in the mouse, and suggests that they may have similar roles in other hosts, including humans.

Previous studies have shown that Gbp1, Gbp2, Gbp3, Gbp6, Gbp7, and Gbp9 are recruited to PV containing *T. gondii* in IFN-γ-activated MEFs or RAW 264.7 macrophages and that the type I BK strain avoids recruitment of a subset of these (i.e. Gbp1, Gbp2, Gbp3, and Gbp6) [30]. Similar findings were reported using MEFs transfected with an epitope-tagged version of Gbp1 that was recruited to PV containing type II (Pru strain) and type III (CEP strain), but not type I (RH strain) parasites [31]. This later study also made use of transgenic type III strain parasites that express the type 1 allele of ROP18, which enhances virulence, and found that this resulted in decreased recruitment of Gbp1 [31]. Here, we confirm and extend these findings by showing that the virulent type I GT-1 strain blocks recruitment of Gbp1, while PV containing avirulent type III (CTG) parasites accumulated this effector protein. We also show that the ability of ROP18 to confer protection on the normally susceptible type III strain CTG depends on the kinase activity, as parasites expressing a kinase dead form of ROP18 (CTG+ROP18 D/A) accumulated Gbp1 at levels slightly higher than CTG alone. Further, type I mutants lacking either ROP18 or ROP5 were also susceptible to recruitment of Gbp1, a phenotype that was fully complemented by re-expression of the respective virulence factors.

ROP18 has previously been shown to affect survival in IFN-γ-activated macrophages, a process that occurs due to selective phosphorylation of IRG proteins, thereby blocking GTPase activity and disrupting vacuole recruitment [24], [25]. In contrast, ROP5 functions by disrupting oligomerization of Irga6 [38], [39], and/or by directly enhancing the catalytic activity of ROP18 [9]. The mechanisms by which the virulent alleles of ROP5 and ROP18 prevent GBP accumulation are presently uncertain but may involve similar functions. Gbp1 and Gbp2 have been shown to form tetramers in response to GTP binding, leading to cooperative activation of GTPase activity [28], [40]. Mutants in the GTP binding domain of Gbp2 that block GTPase activity and alter formation of multimers, disrupts loading onto PV containing susceptible *T. gondii* [40], while similar mutations in Gbp1 impairs control of both *Listeria* and *Mycobacteria* [28]. Whether GBPs are a direct target of ROP18 phosphorylation is presently unknown, although they contain regions similar to the described motif of ROP18 [24]. Further studies designed to elucidate the mechanisms by which ROP5 and ROP18 disrupt GBP recruitment are currently underway.

Previous kinetic studies of IRG recruitment to PV containing susceptible parasites shows that Irgb6 and Irgb10 arrive early, followed by other IRG proteins such as Irga6 [22]. Given their previously established importance in the clearance process, Irga6 [41] and Irgb6 [24] were used here as sentinels of this pathway. Previous studies have shown that PV containing susceptible parasites are rapidly stripped of their membrane and the parasite within the vacuole is digested [22]; hence, the percentage of IRG positive vacuoles at any given time point reflects only a portion of the parasites that ultimately are destroyed in the overnight clearance assay. Our findings are consistent with this model in that we observed efficient loading of Irgb6 at early time points (30 min) and the level increased slightly over the first 2 hr, consistent with previous reports [23], [24]. Others have reported that Irgb6 accumulation to vacuoles containing susceptible parasites (i.e. CTG) is decreased in cells lacking the Gbp^chr3^ locus [32]. Here, we show that the initial recruitment of Irgb6 is normal at 30 min, but the retention of this marker is impaired with fewer PV remaining positive at 2 hr. Combined with the finding that Irgb6 co-precipitates with Gbp1–5 [32], this suggests that accumulation of GBPs is associated with stabilization of IRGs and their retention on the vacuole membrane. Whether GBPs contribute directly to membrane blebbing and physical disruption of the PVM, or modulate the function of other effectors at this interface, remains to be demonstrated by further studies. Together these data suggests that destruction of the PV in response to IFN-γ requires a sequential recruitment of both IRG and GBP proteins, ultimately resulting in membrane disruption.

Autophagy proteins also participate in cell autonomous clearance of parasites, although these may be due to indirect effects on the homeostasis of effectors such as IRGs and GBPs. Utilizing Atg5-deficient MEFs, we examined the localization of Gbp1 in IFN-γ-activated cells. Immunofluorescence and electron microscopy showed that Atg5-deficient cells have large membrane-associated aggregates of Gbp1 in their cytoplasm and that these co-localize with the active GTP-bound form of Irga6, ubiquitin, and the autophagy adaptor protein p62. Although, they do not co-localize with LAMP1 in Atg5^−/−^ cells, they do proceed to autophagolysosomes in wild type cells and Gbp1 co-immunoprecipitates p62 from cell lysates in a manner that does not require the p62 ubiquitin-binding domain [28]. Similarly, previous studies have also shown that in the absence of IRGM proteins, both IRG and GBP proteins form co-aggregates that colocalize with the adaptor p62 and the autophagy protein LC3 [36]. Collectively, these results suggest that autophagy is necessary for removal of aggregates that spontaneously form in wild type cells, and which are degraded by a classical autophagy pathway that terminates in lysosomal fusion.

The aberrant localization of Gbp1 in Atg5-deficient cells provided an opportunity to examine the functional consequences of this disruption on parasite clearance in macrophages that were selectively deleted for Atg5 using LysMcre recombination. Recruitment of both Irgb6 and Gbp1 to susceptible parasites (RHΔ*ku80*Δ*rop18* and RHΔ*ku80*Δ*rop5*) was significantly reduced in BMM lacking Atg5. Additionally, clearance of these susceptible parasite strains was almost completely reversed in Atg5-deficient BMM. These effects are most likely due to the aberrant localization of IRGs and GBPs that occurred in the absence of Atg5. Improper localization of IRGs and GBPs may be due to a requirement for autophagy to remove misfolded aggregates that otherwise drive inappropriate activation. Interestingly, we have observed a similar requirement for Atg7 and Atg16 in cell-autonomous control of *T. gondii* in IFN-γ-activated BMM *in vitro*, although this requirement may not extend to all mediators of the autophagy pathway (S. Hwang and H.W. Virgin, unpublished). Alternatively, the requirement for Atg5 may not reflect a classical autophagy degradation pathway, but rather a role in the delivery of effectors to pathogen containing vacuoles, as suggested by the membranous accumulations that occur in the proximity of PV that are targeted for destruction (Figure 2, 3 present study, and [23], [28]). Consistent with this, GBPs are localized to membrane vesicles within the cytoplasm of host cells, and several contain a C-terminal CaaX box allowing for isoprenylation that facilitates interaction with host cell membranes [28], [30]. Regardless of the exact role of Atg proteins, this illustrates the critical balance of control of these two families of effectors, which are maintained in an inactive state in order to avoid damage to host membranes, yet need to be readily mobilized for pathogen clearance.

Previous work has shown that a genetic knockout of the Gbp^chr3^ locus increases susceptibility to a type II strain *T. gondii*, which has an intermediate level of virulence in the mouse [32]. Analyzing the phenotype of this mutant is complicated due to the simultaneous disruption of five intact genes as well as a truncated form of Gbp2. Characterization of this mouse revealed that it has normal expansion of CD4^+^ and CD8^+^ T cells, and produces normal levels of IL-12 and IFN-γ following infection with *T. gondii* [32]. Macrophages from these Gbp^chr3−/−^ mice also produce normal levels of O_2_^−^ and NO in response to IFN-γ [32]. Despite having intact responses, IFN-γ-activated BMM from these animals show specific defects in the control of *T. gondii* replication (stasis), as well as ability to clear parasites (clearance) *in vitro*. This former phenotype suggests that GBPs play a role in the NO-mediated stasis that impairs parasite replication, while the later defect is due to decreased recruitment of Irgb6 and an inability to vesiculate and destroy PV [32]. Reconstitution experiments suggested that Gbp 1, 5 and 7 contribute to this clearance defect *in vitro*, although the over-expression strategy used in this study complicates the interpretation of these findings.

In the present work, we have examined a single knockout of Gbp1 to address the role of this protein in resistance to *T. gondii*. We observed that both the ability to induce stasis and the capacity for clearance of susceptible parasites *in vitro* was greatly reduced in IFN-γ-activated macrophages from Gbp1^−/−^ *vs.* wild type mice. Hence, the phenotype of BMM from the Gbp1^−/−^ mouse partially recapitulates the two major defects seen in cells from the Gbp^chr3^ deletion mouse when tested *in vitro*. Greater susceptibility was also seen *in vivo*, with Gbp1^−/−^ mice succumbing to infection earlier with Δ*rop18* parasites, which are partially attenuated in virulence towards wild type mice. Furthermore, Gbp1 was important for resistance to challenge with an intermediate virulence, type II strain of *T. gondii*, with the majority of mice succumbing after the initial acute infection. Surviving Gbp1^−/−^ mice showed elevated CNS pathology consistent with encephalitis as the cause of death. Recent studies using a single gene deletion of Gbp2^−/−^ also reported loss of the ability to control replication *in vitro* and increased susceptibility to type II strain challenge during the chronic phase [34]. The increased chronic susceptibility of Gbp1^−/−^ or Gbp2*^−/−^* mice is similar to that previously described for Nos2^−/−^ mice, which lack inducible nitric oxide synthase and hence produce lower levels of NO [37]. However, Gbp^chr3−/−^ mice produce normal levels of NO when stimulated with IFN-γ, despite losing the capacity to control parasite replication *in vitro*. Hence, the increased susceptibility in Gbp1^−/−^ or Gbp2*^−/−^* mice to chronic infection may reflect reduced NO production locally within the CNS, or result from impaired clearance via the IRG or GBP pathways, leading to higher chronic burdens of infection.

GBPs have recently been implicated in resistance to several pathogens in the mouse and yet they likely play overlapping yet different roles with respect to individual pathogens [26]. Gbp1^−/−^ and Gbp5^−/−^ mice were first shown to be important for control of *Listeria* and *Mycobacteria* infections [28], [33]. Individual deletion of Gbp2 [34] or Gbp1 (present report), renders mice susceptible to *T. gondii*, while additional genes within the Gbp^chr3^ locus may likewise contribute since the phenotype of this deletion is more severe than any of the single mutants [32]. In contrast, individual deletion of Gbp2ps, an alternatively spliced variant, or of Gbp5 does not affect susceptibility to i.p. challenge with type II ME49 parasites [32]. The diversity of GBPs may be an adaptation to target different intracellular pathogens: availability of additional gene disruptants will facilitate further testing of the role of individual GBPs in resistance to a variety of pathogens. As well, the diverse nature of pathogens affected by GBPs suggest that they play indirect roles in affecting delivery of other effectors, as reported previously for control of intracellular bacterial pathogens [28], or the retention of Irgb6 to *T. gondii* vacuoles as shown here, and reported previously [32].

Although studies on GBPs conducted to date have focused on their role in resistance in the mouse, this family of proteins is highly conserved in vertebrates [42] and even some protochordates [33]. Several studies have examined their participation in resistance to infection in humans. Human GBP1 has been shown to contribute to anti-viral activity against vesicular stomatitis virus and encephalomyocarditis virus [43] and hepatitis C [44], and recent studies implicate hGBP3 in resistance to influenza [45]. Previous work examining the role of GBPs in human cells show that GBP1 is recruited to the inclusion membrane of Chlamydia and over-expression contributes to smaller inclusion size [46]. The conservation of this family of proteins in humans and other higher order mammals [26] suggests that the GBPs play a more widespread role in resistance to infection.

## Materials and Methods

### Parasite strains and culture

Type I (GT-1), type III transfection control (CTG Ble), type III parasites expressing ROP18 clone V1 (CTG+ROP18) and type III parasites expressing a kinase-dead ROP18 clone L1 (CTG+ROP18 D/A) were described previously [8]. Type I RHΔ*ku80*Δ*Hx* (RHΔ*ku80*) parasites described previously [47], were used here as wild type. Type I parasites lacking ROP18 (RHΔ*ku80*Δ*rop18*) [24] and the complemented strain (RHΔ*ku80*Δ*rop18/*ROP18) [35], were described previously. A type I parasite strain lacking ROP5, (RHΔ*ku80*Δ*rop5*) and the complemented strain (RHΔ*ku80*Δ*rop5*/ROP5) were described previously [35]. Luciferase expressing parasites were generated by transfection of parasites with pClickluc, as described previously [48] and isolation of single cell clones. Type II Prugnaud strain parasites expressing firefly luciferase and GFP (PRU-Luc-GFP) were provided by John Boothroyd (Stanford University). Parasites were cultured in human foreskin fibroblasts grown in DMEM supplemented with 10% fetal bovine serum (HyClone, Thermo Scientific, Rockford, IL), 2 mM glutamine, 10 mM HEPES pH 7.5 and 20 µg/ml gentamicin at 37°C under 5% CO_2_. For all experiments, parasites were allowed to naturally egress and harvested shortly thereafter as described previously [49].

### Ethics statement

All animal experiments were conducted according to the U.S.A. Public Health Service Policy on Humane Care and Use of Laboratory Animals. Animals were maintained in an AAALAC-approved facility and all protocols were approved by the Institutional Care Committee (School of Medicine, Washington University in St. Louis).

### Animal studies

CD-1 and C57BL/6 mice were obtained from Charles River Laboratory (Wilmington, MA). Gbp1^−/−^ mice, originally derived on a 129 background and backcrossed 6–8 times to C57BL/6, were provided by the MacMicking laboratory as described previously [28], and bred locally. *Atg5^flox/flox^* (control) mice and *Atg5^flox/flox^*+LysMcre mice (8–12 weeks old) provided by the Virgin laboratory were bred locally and genotyped as described [23]. Irgm3^−/−^ mice on a C57/BL6 background were provided by Greg Taylor (Taylor et al., 2000), and bred locally. For *in vivo* challenges with type I strains, 8–12 week old female C57BL/6 and Gbp1^−/−^ mice were infected s.c. with 100 freshly egressed parasites and survival was monitored for 30 days as described [8], [49]. For *in vivo* challenges with PRU-Luc-GFP parasites, 8–12 week old (male and female, matched per group) C57BL/6 and Gbp1^−/−^ mice were infected i.p. with either 500 or 1,000 freshly egressed parasites and survival was monitored for 60 days.

### Cell culture

Bone marrow-derived macrophages (BMMs) and RAW 264.7 macrophages were cultured as described previously [24]. Immortalized MEFs were cultured in DMEM supplemented with L-glutamine and 10% FBS. Where indicated, cells were activated by treatment with murine recombinant IFN-γ (R&D Systems, Minneapolis, MN) and LPS (*E. coli* O55:B5) (Sigma-Aldrich, St. Louis, MO) for 18–24 hr before use.

### Antibodies

The PVM was localized with mouse mAb anti-GRA5 Tg17–113 [50], or rabbit polyclonal sera to GRA7 [51]. Intracellular *T. gondii* parasites were localized with either mouse mAb anti-SAG1 DG52 or rabbit polyclonal sera to RH strain tachyzoites. Gbp1 and was localized with rabbit polyclonal sera raised against peptides specific to each protein [30]. Irgb6 was localized with rabbit anti-Irgb6 [52] or goat anti-TGTP (Santa Cruz Biotechnology, Dallas, TX) as indicated. Irga6 was localized with mouse mAb 10D7, which recognizes the GTP bound form. p62 was localized with guinea pig polyclonal antibody specific to the C-terminus (Progen, Heidelberg, Germany). Ubiquitin was localized with mouse mAb FK2 (EMD Millipore Corporation, Billerica, MA). LAMP1 was localized with rat mAb ID4B, obtained from the Developmental Studies Hybridoma Bank (http://dshb.biology.uiowa.edu). Secondary antibodies conjugated to Alexa Fluor 488 or 594 (Invitrogen, Grand Island, NY) were used for detection by immunofluorescence.

### Immunofluorescence assays

Cells for immunofluorescence were fixed in 4% formaldehyde, permeabilized with 0.05% saponin, and stained using primary and secondary antibodies as described previously [24]. Samples were visualized using a Zeiss Axioskop 2 MOT Plus microscope equipped for epifluorescence and using a 63× PlanApochromat lens, N.A. 1.40 (Carl Zeiss, Inc., Thornwood, NY). Images were acquired with an AxioCam MRm camera (Carl Zeiss, Inc.) using Axiovision v4.6, and processed using similar linear adjustments for all samples in Photoshop CS4 v9. For deconvolution, images were acquired as above using automatic Z-stack acquisition in Axiovision and deconvolved using the nearest neighbor algorithm.

### Cellular recruitment assays

To examine the distribution of host cellular proteins, samples were fixed between 30 min to 2 hr post infection (see legends for specific details). After staining with appropriate primary and secondary antibodies, recruitment was determined by first visualizing the parasitophorous vacuole or parasite marker, then assessing whether there was an accumulation of host protein around each parasite. The percentage of positive parasites was determined from 10 representative fields that were examined at 63×.

### Intracellular clearance and replication assays

To examine intracellular clearance, IFN-γ-activated macrophages were infected with freshly egressed parasites, and then either fixed at 30 min post infection, or returned to culture in complete medium for 20 hr. Clearance was assessed by comparing the percentage of cells infected at 30 min vs. those remaining after 20 hr, as described previously [24]. For 3 biological replicates, 10 fields were counted on each of three coverslips that were examined at 63×. To examine intracellular replication, BMM were activated with IFN-γ and infected with wild type parasites for 30 min, washed to remove extracellular parasites, and recultured overnight in complete medium. At 20 hr post infection, cells were fixed, permeabilized as above, and visualized using mAb to SAG1 (DG52) directly conjugated to Alexa Fluor 594. The number of parasites per vacuole was determined from counting 100 vacuoles per sample from three separate coverslips. In general, 15–30% of cells were singly infected at 30 min. In order to combine experiments with different initial infection rates, the data were normalized by expressing the infection rate at 20 hr as a percentage of the infection rate at 30 min.

### Transmission electron microscopy

Samples for EM were activated with IFN-γ and LPS for 18–24 hr. Where indicated, cells were infected with freshly egressed parasites for 30 min, washed three times with PBS then fixed at 2 and 6 hr post infection. For ultrastructural analysis, cells were fixed in 2% paraformaldehyde/2.5% glutaraldehyde (Polysciences Inc., Warrington, PA) in 100 mM phosphate buffer, pH 7.2 for 1 hr at room temperature, processed and examined as described previously [23], [24]. For immuno-EM, cells were fixed in 4% paraformaldehyde/0.05% glutaraldehyde (Polysciences Inc.,) in 100 mM PIPES/0.5 mM MgCl_2_, pH 7.2 for 1 hr at 4°C, and processed as described previously [24]. Sections were stained with mouse anti-protein disulfide isomerase (Enzo Life Sciences, Inc. Farmington, NY) and rabbit anti-Gbp1 antibodies for 1 hr at room temperature, followed by gold-conjugated secondary antibodies (Jackson ImmunoResearch Laboratories, Inc., West Grove PA). Sections were stained and viewed with a JEOL 1200EX transmission electron microscope (JEOL USA Inc., Peabody, MA), as described previously [24]. Parallel controls omitting primary antibodies were consistently negative at the concentration of colloidal gold conjugated secondary antibodies used in these studies.

### Histological studies

Animals were sacrificed at 60 days post infection; the brain was removed and fixed in 10% neutral- buffered formalin. Tissues were dehydrated in ethanol and embedded in paraffin, and 5 micron sections were stained with hematoxylin and eosin (H&E). Sections were evaluated by veterinary pathologist in the Department of Comparative Animal Medicine, Washington University.

### Statistics

Statistical analyses were conducted using Microsoft Excel and PRISM. Excel results were compared using the Student's *t* tests performed under the assumption of equal variance and with a two-tailed test where *P*≤0.05 was considered significant. Survival statistics were compared using log-rank and Gehan-Breslow-Wilcoxin tests in PRISM. Data were graphed as means ± standard deviation of the population (S.D.P.), or as standard deviation (S.D.), as noted.

## Supporting Information

Figure S1 Localization of Gbp1 aggregates with ubiquitin, p62, or LAMP1 in wild type murine embryonic fibroblasts (MEFs). (A–C) Immunofluorescence of untreated wild type (Atg5 WT) MEFs activated with IFN-γ and LPS (100 U/ml and 0.1 ng/ml respectively) for 18 hours. Gbp1 localized with rabbit polyclonal sera followed by goat anti-rabbit IgG conjugated to Alexa Fluor 488. (A) Ubiquitin was localized with mAbFK2 followed by goat anti-mouse IgG conjugated to Alexa fluor 594. (B) p62 was localized with guinea pig polyclonal sera followed by goat anti-guinea pig IgG conjugated to Alexa Fluor 594. (C) LAMP1 was localized with rat mAB 1D4B followed by goat anti-rat 1gG conjugated to Alexa fluor 594. (D–E) Wild type Atg5 MEFs were treated with 1 µM Bafilomycin (Invivogen, San Diego, CA) concurrent with IFN-γ and LPS activation (100 U/ml, 0.1 ng/ml respectively) for 18 hr. (D) Ubiquitin was localized with mAb FK2 followed by goat anti-mouse IgG conjugated to Alexa fluor 594. (E) p62 was localized with guinea pig polyclonal sera followed by goat anti-guinea pig IgG conjugated to Alexa Fluor 594. Scale bar = 5 µm, similar scale for all images. In all panels, cells were permeabilized with 0.05% saponin, blocked with 5% FBS, 5% normal goat serum in 0.05% saponin, and washed with 1% normal goat serum in 0.01% saponin. Samples were stained with primary and secondary antibodies and mounted in ProLong Gold antifade reagent with DAPI (Molecular Probes, Eugene, OR), as described in the methods. Samples were visualized using a Zeiss Axioskop 2 MOT Plus microscope equipped for epifluorescence and using a 63× PlanApochromat lens, N.A. 1.40 (Carl Zeiss, Inc., Thornwood, NY). Images were acquired with an AxioCam MRm camera (Carl Zeiss, Inc.) using Axiovision v4.6. Images in panels A,B, D, and E are wide field epifluorescence. Images in C were acquired using automatic Z-stack acquisition in Axiovision and deconvolved using the nearest neighbor algorithm. A representative central slice was exported to Photoshop and adjusted using similar settings.
(TIF)

Figure S2 Ultrastructural features of the parasitophorous vacuole membrane of parasites that have undergone vacuole blebbing, stripping and death in the cytoplasm of IFN-γ-activated bone marrow derived macrophages from wild type mice infected with ROP5-deficient (RH*Δku80Δrop5*) or ROP18-deficient (RH*Δku80Δrop18*) parasites. (A–C) ROP5-deficient parasites with vacuole membranes that showed blebbing (A), vacuole stripping (B) and parasite death (C). Similar ultrastructrual features are seen for ROP18-deficient parasites (D–F). Scale bars = 500 nm. Samples for EM were activated with 50 U/ml IFN-γ and 10 ng/ml LPS for 18–24 hr. Cells were infected with freshly egressed parasites for 30 min, washed three times with PBS then fixed at 2 to 6 hr post infection. For ultrastructural analysis, cells were fixed in 2% paraformaldehyde/2.5% glutaraldehyde (Polysciences Inc., Warrington, PA) in 100 mM phosphate buffer, pH 7.2 for 1 hr at room temperature, processed and examined as described previously [23], [24]
(TIF)
