# Supplementary figures and images for: Guanylate-binding Protein 1 (Gbp1) Contributes to Cell-autonomous Immunity against Toxoplasma gondii
Source: PLoS Pathog. 2013 Apr 25;9(4):e1003320. doi: 10.1371/journal.ppat.1003320 (PMC3635975; doi:10.1371/journal.ppat.1003320)

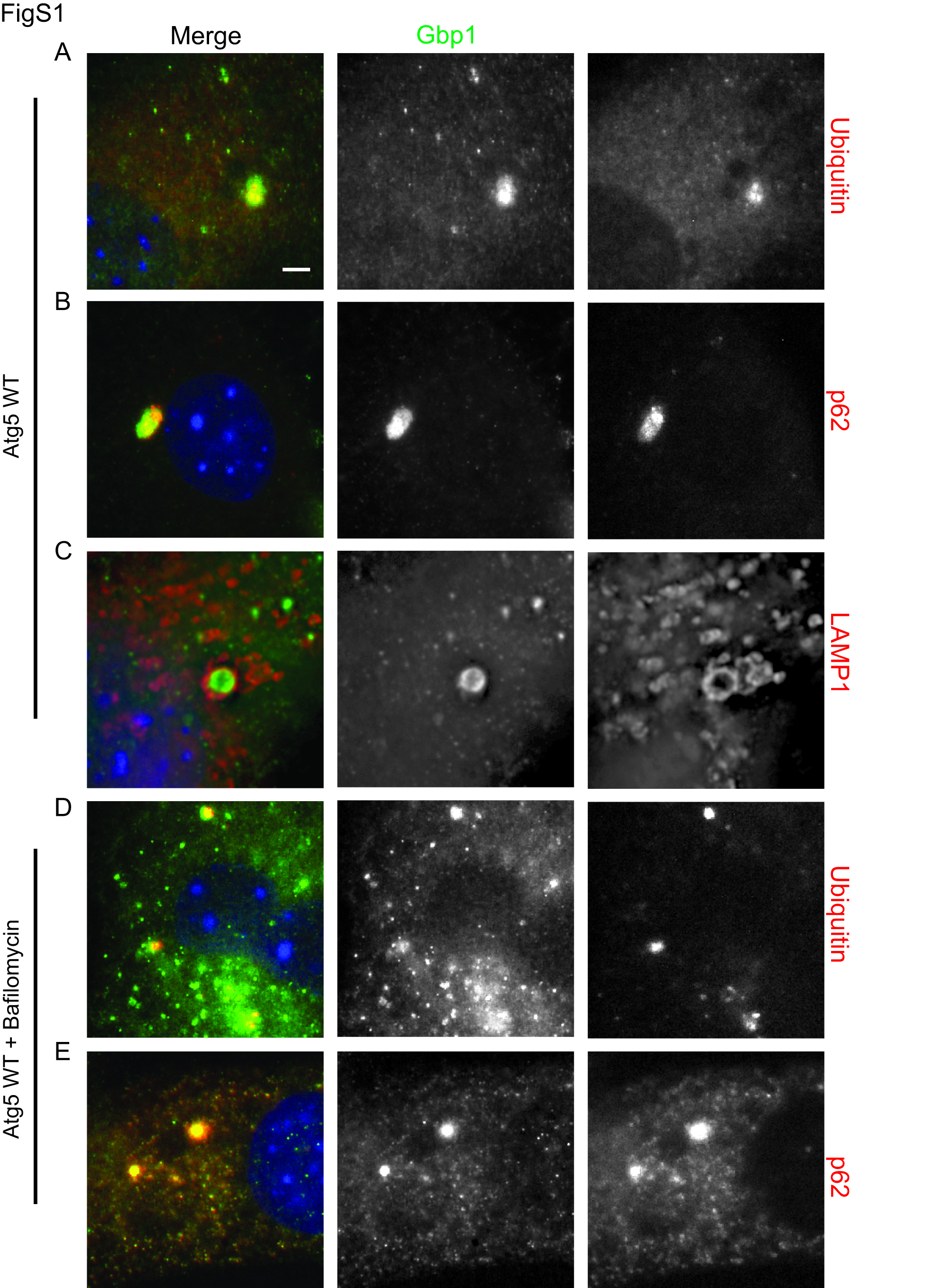

Supplement: Figure S1 — Localization of Gbp1 aggregates with ubiquitin, p62, or LAMP1 in wild type murine embryonic fibroblasts (MEFs). (A–C) Immunofluorescence of untreated wild type (Atg5 WT) MEFs activated with IFN-γ and LPS (100 U/ml and 0.1 ng/ml respectively) for 18 hours. Gbp1 localized with rabbit polyclonal sera followed by goat anti-rabbit IgG conjugated to Alexa Fluor 488. (A) Ubiquitin was localized with mAbFK2 followed by goat anti-mouse IgG conjugated to Alexa fluor 594. (B) p62 was localized with guinea pig polyclonal sera followed by goat anti-guinea pig IgG conjugated to Alexa Fluor 594. (C) LAMP1 was localized with rat mAB 1D4B followed by goat anti-rat 1gG conjugated to Alexa fluor 594. (D–E) Wild type Atg5 MEFs were treated with 1 µM Bafilomycin (Invivogen, San Diego, CA) concurrent with IFN-γ and LPS activation (100 U/ml, 0.1 ng/ml respectively) for 18 hr. (D) Ubiquitin was localized with mAb FK2 followed by goat anti-mouse IgG conjugated to Alexa fluor 594. (E) p62 was localized with guinea pig polyclonal sera followed by goat anti-guinea pig IgG conjugated to Alexa Fluor 594. Scale bar = 5 µm, similar scale for all images. In all panels, cells were permeabilized with 0.05% saponin, blocked with 5% FBS, 5% normal goat serum in 0.05% saponin, and washed with 1% normal goat serum in 0.01% saponin. Samples were stained with primary and secondary antibodies and mounted in ProLong Gold antifade reagent with DAPI (Molecular Probes, Eugene, OR), as described in the methods. Samples were visualized using a Zeiss Axioskop 2 MOT Plus microscope equipped for epifluorescence and using a 63× PlanApochromat lens, N.A. 1.40 (Carl Zeiss, Inc., Thornwood, NY). Images were acquired with an AxioCam MRm camera (Carl Zeiss, Inc.) using Axiovision v4.6. Images in panels A,B, D, and E are wide field epifluorescence. Images in C were acquired using automatic Z-stack acquisition in Axiovision and deconvolved using the nearest neighbor algorithm. A representative central slice was exported to [file ppat.1003320.s001.tif]

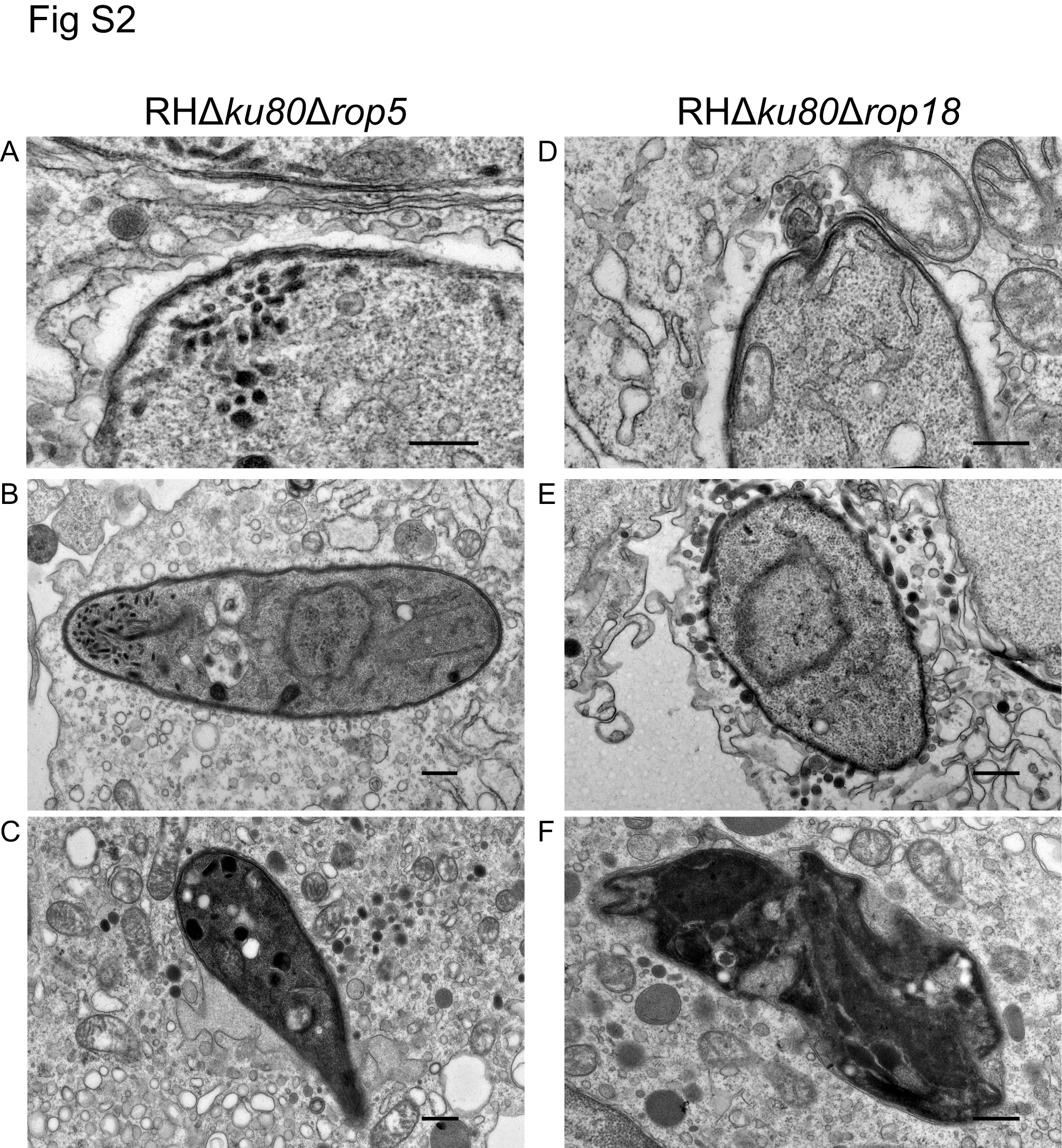

Supplement: Figure S2 — Ultrastructural features of the parasitophorous vacuole membrane of parasites that have undergone vacuole blebbing, stripping and death in the cytoplasm of IFN-γ-activated bone marrow derived macrophages from wild type mice infected with ROP5-deficient (RHΔku80Δrop5) or ROP18-deficient (RHΔku80Δrop18) parasites. (A–C) ROP5-deficient parasites with vacuole membranes that showed blebbing (A), vacuole stripping (B) and parasite death (C). Similar ultrastructrual features are seen for ROP18-deficient parasites (D–F). Scale bars = 500 nm. Samples for EM were activated with 50 U/ml IFN-γ and 10 ng/ml LPS for 18–24 hr. Cells were infected with freshly egressed parasites for 30 min, washed three times with PBS then fixed at 2 to 6 hr post infection. For ultrastructural analysis, cells were fixed in 2% paraformaldehyde/2.5% glutaraldehyde (Polysciences Inc., Warrington, PA) in 100 mM phosphate buffer, pH 7.2 for 1 hr at room temperature, processed and examined as described previously [23], [24] (TIF) [file ppat.1003320.s002.tif]
